# A Small-Molecule Modulator of Metal Homeostasis in Gram-Positive Pathogens

**DOI:** 10.1128/mBio.02555-20

**Published:** 2020-10-27

**Authors:** Lillian J. Juttukonda, William N. Beavers, Daisy Unsihuay, Kwangho Kim, Gleb Pishchany, Kyle J. Horning, Andy Weiss, Hassan Al-Tameemi, Jeffrey M. Boyd, Gary A. Sulikowski, Aaron B. Bowman, Eric P. Skaar

**Affiliations:** a Vanderbilt Institute for Infection, Immunology and Inflammation, Vanderbilt University Medical Center, Nashville, Tennessee, USA; b Chemical Synthesis Core, Vanderbilt University, Nashville, Tennessee, USA; c Department of Biological Chemistry and Molecular Pharmacology, Harvard Medical School, Boston, Massachusetts, USA; d Vanderbilt Brain Institute, Department of Pediatrics, Vanderbilt University School of Medicine, Nashville, Tennessee, USA; e Department of Biochemistry and Microbiology, Rutgers, the State University of New Jersey, New Brunswick, New Jersey, USA; f Department of Chemistry, Vanderbilt University, Nashville, Tennessee, USA; g Department of Pathology, Microbiology, and Immunology, Vanderbilt University Medical Center, Nashville, Tennessee, USA; University of Rochester

**Keywords:** MRSA, *Staphylococcus aureus*, antibiotics, cobalt, copper, manganese, metalloregulation

## Abstract

Metals are essential nutrients that all living organisms acquire from their environment. While metals are necessary for life, excess metal uptake can be toxic; therefore, intracellular metal levels are tightly regulated in bacterial cells. Staphylococcus aureus, a Gram-positive bacterium, relies on metal uptake and metabolism to colonize vertebrates. Thus, we hypothesized that an expanded understanding of metal homeostasis in S. aureus will lead to the discovery of pathways that can be targeted with future antimicrobials. We sought to identify small molecules that inhibit S. aureus growth in a metal-dependent manner as a strategy to uncover pathways that maintain metal homeostasis. Here, we demonstrate that VU0026921 kills S. aureus through disruption of metal homeostasis. VU0026921 activity was characterized through cell culture assays, transcriptional sequencing, compound structure-activity relationship, reactive oxygen species (ROS) generation assays, metal binding assays, and metal level analyses. VU0026921 disrupts metal homeostasis in S. aureus, increasing intracellular accumulation of metals and leading to toxicity through mismetalation of enzymes, generation of reactive oxygen species, or disruption of other cellular processes. Antioxidants partially protect S. aureus from VU0026921 killing, emphasizing the role of reactive oxygen species in the mechanism of killing, but VU0026921 also kills S. aureus anaerobically, indicating that the observed toxicity is not solely oxygen dependent. VU0026921 disrupts metal homeostasis in multiple Gram-positive bacteria, leading to increased reactive oxygen species and cell death, demonstrating the broad applicability of these findings. Further, this study validates VU0026921 as a probe to further decipher mechanisms required to maintain metal homeostasis in Gram-positive bacteria.

## Abstract (precis)

Staphylococcus aureus is a leading agent of antibiotic-resistant bacterial infections in the world. S. aureus tightly controls metal homeostasis during infection, and disruption of metal uptake systems impairs staphylococcal virulence. We identified small molecules that interfere with metal handling in S. aureus to develop chemical probes to investigate metallobiology in this organism. Compound VU0026921 was identified as a small molecule that kills S. aureus both aerobically and anaerobically. The activity of VU0026921 is modulated by metal supplementation, is enhanced by genetic inactivation of Mn homeostasis genes, and correlates with increased cellular reactive oxygen species. Treatment with VU0026921 causes accumulation of multiple metals within S. aureus cells and concomitant upregulation of genes involved in metal detoxification. This work defines a small-molecule probe for further defining the role of metal toxicity in S. aureus and validates future antibiotic development targeting metal toxicity pathways.

## INTRODUCTION

Bacterial infections remain a significant cause of morbidity and mortality worldwide, and antibiotic resistance has become commonplace amid a decades-long drought in antibiotic development (1, 2). Most antibiotics brought to market are derived from existing antibiotic classes and may be rendered obsolete through shared antibiotic resistance mechanisms (3). Therefore, therapeutic development for antibiotic-resistant bacterial pathogens has been named a priority by numerous public health organizations (4, 5).

Staphylococcus aureus is a major threat to human health and one of the most common causes of bacterial infection in the world (6). Effective antibiotic treatment prevents morbidity and mortality due to S. aureus infections. However, methicillin-resistant S. aureus (MRSA) strains are resistant to multiple classes of beta-lactam antibiotics (7). MRSA surgical site wounds and central line-associated bloodstream infections in the United States alone cost more than $1.3 billion per year (8). The Centers for Disease Control and Prevention labeled MRSA a “serious threat,” and the World Health Organization categorized the need for new antibiotics against MRSA as a “high priority” (4, 5). Investigations into processes essential for S. aureus fitness may reveal fruitful avenues for the development of novel antibiotics.

The transition metals iron (Fe), manganese (Mn), and zinc (Zn) serve as structural elements within macromolecules and as essential cofactors for enzymes and are thus necessary for S. aureus survival. However, transition metals are toxic when present in excess as they compete with cognate metals for protein binding pockets or transport (9, 10). Furthermore, redox-active metals can generate reactive oxygen species (ROS) through Fenton chemistry (11, 12). Consequently, transition metal homeostasis is imperative for pathogen success during infection. Vertebrate hosts limit bacterial access to essential metals in a process termed “nutritional immunity,” and a parallel process exists for intoxicating invaders with highly reactive metals (13–16). Bacteria strictly control metal levels such that metals are available to support biological processes while preventing toxicity. Metal levels are maintained through a balance of import and efflux systems as well as metal-sequestering molecules within the cell (9). During infection, maintaining appropriate metal ratios is essential for bacterial survival and virulence. We predict that metal homeostasis is a process that can be exploited by targeted antimicrobial therapy during infection.

Given the importance of metal acquisition and homeostasis in bacterial physiology, several studies have investigated the potential of developing antimicrobials that act by disrupting metal homeostasis (17). These approaches have included gallium-based antimicrobials, which disrupt Fe import and homeostasis (18, 19); gallium porphyrin molecules that disrupt heme-iron import and homeostasis (20); and metal chelators (21, 22). However, these strategies have heretofore relied on imposing metal starvation, whereas exploitation of metal toxicity is less common.

Here, we present the identification of a small molecule that causes metal accumulation and toxicity in S. aureus. A library of compounds previously identified to alter Mn transport in mammalian cells (23) was screened for the ability to differentially inhibit growth of S. aureus lacking high-affinity Mn uptake machinery. Compound VU0026921 (‘921) decreases S. aureus viability, exhibiting enhanced activity in an S. aureus strain lacking high-affinity Mn import systems. Supplementation with divalent transition metals modulates the antimicrobial activity of VU0026921, and cellular ROS levels are inversely correlated with the ability of each metal-VU0026921 combination to kill S. aureus. However, the activity of VU0026921 is not mediated solely through ROS generation because antioxidants provide only partial protection from VU0026921 killing, and the molecule kills S. aureus both aerobically and anaerobically. RNA sequencing revealed that VU0026921 causes upregulation of multiple metal exporters and metal homeostasis machinery, suggesting that VU0026921 induces a metal toxicity response in S. aureus. Indeed, VU0026921 binds cobalt (Co), copper (Cu), Fe, Mn, and Zn, leading to increases in cellular Zn, Fe, and Cu following compound exposure as uncovered by elemental mass spectrometry. Cotreatment with Mn and VU0026921 led to increases in cellular Mn and abrogated accumulation of other metals, suggesting that intracellular Mn maintains cellular viability. Additionally, the exogenous addition of Cu exacerbates the killing of S. aureus by VU0026921, while the exogenous addition of Co is more protective than Mn. VU0026921 has low toxicity in Gram-negative species but kills all of the S. aureus strains and other Gram-positive pathogens tested. Taken together, this work reports the discovery of a small molecule that dysregulates metal homeostasis in S. aureus and other Gram-positive pathogens, causing substantial toxicity to this organism, and facilitates future elucidation of metal dysregulation as an antistaphylococcal treatment.

## RESULTS

### Identification of a small molecule that kills S. aureus.

**FIG 1:**
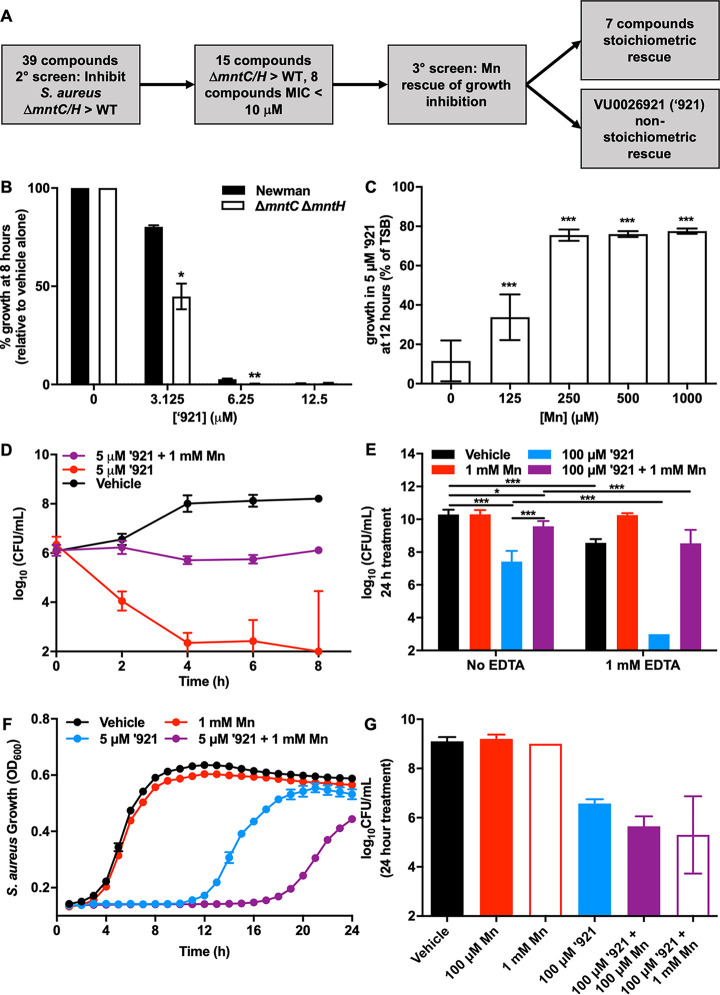
Identification of a small molecule that is antimicrobial to S. aureus. (A) Workflow for identifying compounds of interest. (B) Percent growth inhibition by treatment with the indicated concentrations of compound ‘921 relative to vehicle control for S. aureus Newman or Δ*mntH/C* strain. Data are mean ± standard deviation from duplicate measurements. Statistical significance was determined by *t* test where * = *P < *0.05 and ** = *P < *0.01. (C) Relative growth at 12 h with 5 μM ‘921 ± MnCl_2_ is graphed as % of growth without compound or MnCl_2_. Data are mean ± standard deviation from triplicate measurements acquired on two separate days and combined (*n* = 6). Statistical significance was determined by one-way analysis of variance (ANOVA) with Dunnett’s multiple-comparison test comparing each concentration of Mn to no-Mn control where *** = *P < *0.001. (D) CFU recovered at 2-h intervals from growth curve of S. aureus Newman treated with vehicle, 5 μM ‘921, or 5 μM ‘921 + 1 mM MnCl_2_ over time. Data are mean ± standard deviation from quadruplicate measurements. (E) CFU recovered following 24-h treatment of mid-exponential-phase S. aureus Newman cultures with the indicated combinations of vehicle, 1 mM MnCl_2_, 100 μM ‘921, and 1 mM EDTA. Data are mean ± standard deviation from triplicate measurements acquired on two separate days combined (*n* = 6). Statistical significance was determined by one-way ANOVA with Sidak’s multiple-comparison test comparing each concentration of Mn to no-Mn control where * = *P < *0.05 and *** = *P < *0.001. Experiments in panels F and G were performed in an anaerobic chamber. (F) Growth of S. aureus Newman in the presence of vehicle, 1 mM MnCl_2_, 5 μM ‘921, or 5 μM ‘921 + 1 mM MnCl_2_. Data are mean ± standard deviation for triplicate measurements. (G) CFU recovered following treatment of mid-exponential-phase cultures of S. aureus with vehicle, 1 mM or 100 μM MnCl_2_, 100 μM ‘921, or combinations of MnCl_2_ + ‘921 for 24 h. Data are mean ± standard deviation for triplicate measurements.

We sought to identify small molecules that disrupt S. aureus growth in an Mn-dependent manner with the goal of identifying chemical probes for understanding S. aureus metallobiology. We tested a collection of 39 compounds that alter the net uptake of Mn into mammalian cell lines without causing toxicity (23). The compounds were tested in S. aureus Newman and Newman Δ*mntH/C*, a strain that is Mn starved due to deletion of two Mn import systems (24). This strain does not have a growth defect in rich medium but grows poorly under Mn-limited conditions and is hypersusceptible to oxidative stress because of impaired superoxide dismutase activity (24, 25). This strategy was chosen to identify compounds that imposed stress on S. aureus with impaired Mn homeostasis while also selecting for small molecules that did not target the high-affinity Mn import systems. Any compounds discovered that differentially inhibit S. aureus Newman and Δ*mntH/C* presumably are altering intracellular Mn in S. aureus. Eight compounds were identified that are antimicrobial at concentrations of 10 μM or lower, and 7 of those demonstrated growth inhibition that can be reversed by the addition of MnCl_2_ to the medium in stoichiometric ratios, suggesting that they inhibit growth by chelating Mn in the medium (Fig. 1A and also Table S1B in the supplemental material). Compound VU0026921 was selected for further study because it exhibits differential growth inhibition of the Δ*mntH/C* strain relative to the wild type (WT) (Fig. 1B), and reversal of growth inhibition by Mn is not complete and requires 50-fold excess Mn (Fig. 1C). These data suggest that VU0026921 interferes with S. aureus Mn homeostasis or disrupts a process that can be rescued by excess Mn. Moreover, these results imply that the activity of VU0026921 does not occur simply through extracellular Mn limitation, as a substantial excess of Mn is required to reverse the phenotype.

10.1128/mBio.02555-20.4 TABLE S1 (A) MICs for compounds tested for antimicrobial activity against WT and Δ*mntH/C* S. aureus. MIC was defined as minimum concentration of compound that inhibited growth by at least 50% at 8 h. (B) Mn rescue of compound-mediated growth inhibition at 5 μM. *Rescue: concentration of Mn that resulted in 50% increase in S. aureus growth at 8 h. #N/A indicates that compound is not inhibitory to WT at 5 μM. Download Table S1, XLSX file, 0.1 MB. Copyright © 2020 Juttukonda et al. 2020 Juttukonda et al. This content is distributed under the terms of the Creative Commons Attribution 4.0 International license.

### Aerobic antimicrobial activity of VU0026921 is modulated by Mn.

Compound VU0026921 inhibits S. aureus growth in a manner modulated by Mn (Fig. 1C). The S. aureus lag phase is prolonged by treatment with 5 μM VU0026921, and the addition of Mn partially rescues this phenotype (Fig. S1A). To determine whether VU0026921 inhibits growth or decreases cell viability, S. aureus CFU were measured every 2 h in the presence of VU0026921 (Fig. 1D). While CFU increase in the vehicle-treated group, CFU decrease following treatment with VU0026921, and cotreatment of Mn with VU0026921 protects S. aureus from killing by VU0026921. These data demonstrate that excess Mn protects S. aureus from VU0026921-mediated killing. To determine if metal restriction enhances S. aureus susceptibility to VU0026921-mediated killing, S. aureus mid-exponential-phase cultures were cotreated with VU0026921, Mn, and the metal chelator EDTA. Cotreatment with EDTA and VU0026921 decreases S. aureus CFU more than treatment with VU0026921 alone, and the decrease in CFU could be reversed by the addition of Mn (Fig. 1E). Together, these data are consistent with a model in which VU0026921 kills S. aureus through a process that is altered by the availability of Mn in the medium.

10.1128/mBio.02555-20.1 FIG S1 Antimicrobial activity of VU0026921. (A) S. aureus Newman was treated with vehicle, 5 μM ‘921, or 5 μM ‘921 + 1 mM MnCl_2_, and growth was monitored by optical density at 600 nm for 20 h. Data are mean ± standard deviation from triplicate measurements. (B and C) S. aureus Newman was untreated or treated with 50 μM (B) or 3 μM (C) ‘921 that was incubated with shaking in TSB at 37°C for 0, 4, 8, or 24 h prior to the addition of the bacterial inoculum to assess ‘921 compound stability, and growth was monitored by optical density at 600 nm for 24 h. Data are mean ± standard deviation from triplicate measurements. Download FIG S1, TIF file, 2.7 MB. Copyright © 2020 Juttukonda et al. 2020 Juttukonda et al. This content is distributed under the terms of the Creative Commons Attribution 4.0 International license.

We considered the possibility that Mn may rescue S. aureus from VU0026921-mediated killing by detoxifying oxygen radicals. Some antimicrobial molecules cause the generation of ROS, and S. aureus uses Mn to detoxify superoxide in both superoxide dismutase (SOD)-dependent and SOD-independent mechanisms (25–29). VU0026921 inhibits S. aureus growth under anaerobic conditions, demonstrating that oxygen is not required for VU0026921 toxicity (Fig. 1F and G). In contrast to the phenotype seen under aerobic conditions, the addition of Mn does not decrease the anaerobic toxicity of VU0026921 (Fig. 1F and G). These results suggest that VU0026921 may have multiple cellular targets, which is commonly observed for biologically active small molecules (30).

### Isolation of VU0026921-resistant S. aureus strains.

Multiple strategies were employed to attempt to generate resistant strains to identify cellular targets of VU0026921 toxicity. First, selection was attempted by plating S. aureus on solid medium impregnated with VU0026921. Despite multiple iterations using various concentrations, growth conditions, and media, single colonies were never observed; growth occurred in a lawn, no growth occurred, or groups of colonies occurred on the edges of plates. Bacteria isolated from VU0026921-impregnated plates did not have enhanced resistance in the presence of VU0026921 under solid or liquid growth conditions. The second selection strategy employed was serial passage in escalating concentrations of VU0026921 in liquid medium. While growth was observed after incubation in the presence of low concentrations of compound, bacterial isolates from these experiments did not exhibit either transient or permanent enhanced resistance in the presence of VU0026921 under solid or liquid growth conditions. Finally, a third strategy involved screening transposon mutants for enhanced growth in liquid medium containing VU0026921; a pilot screen of approximately 200 known MRSA transposon mutants did not identify any strains with significantly enhanced growth that was reproducible across multiple experimental days, and this strategy was not further pursued. No attempts were made to isolate resistant mutants using chemical mutagenesis or pooled transposon libraries. The fact that VU0026921-resistant strains could not be readily isolated is consistent with the possibility of multiple cellular targets of the compound.

### VU0026921 induces a transcriptional response indicative of metal toxicity.

Seeking insight into cellular processes affected by VU0026921, we performed RNA sequencing on aerobically grown exponential-phase S. aureus Newman following 30-min treatment with 100 μM VU0026921. Exposure to VU0026921 resulted in 298 upregulated and 220 downregulated genes at a cutoff of log_2_|FC| = 2 (Table S2A), demonstrating that VU0026921 dramatically alters the transcriptional profile of S. aureus. A Cluster of Orthologous Groups of proteins (COG) analysis for the up- and downregulated transcripts following VU0026921 treatment (Table S2B) demonstrates that the major upregulated COGs are related to toxin production, posttranslational modification/protein turnover/chaperone functions, and translation while energy production is the major downregulated COG, consistent with the stress that S. aureus experiences upon VU0026921 treatment (31–33). Since cotreatment with Mn rescues S. aureus from VU0026921-mediated toxicity, we also analyzed RNA sequencing results for S. aureus cotreated with VU0026921 and Mn to identify Mn-dependent transcriptional changes. Two hundred sixteen genes are upregulated and 147 genes are downregulated by cotreatment with VU0026921 and Mn compared to treatment with compound alone (Table S3). These changes are VU0026921 dependent, as treatment with MnCl_2_ without compound only downregulates the high-affinity *mntABC* operon, known to be repressed by Mn, and upregulates the Mn exporter *mntE* (34, 35) (Table S4).

10.1128/mBio.02555-20.5 TABLE S2 (A) Transcripts significantly* changed# by treatment with 100 μM ‘921. *Significance was defined as false-discovery rate (FDR) < 0.001. #Defined as |log_2_ fold change| >2. (B) COG analysis of upregulated and downregulated genes in S. aureus upon VU0026921 treatment. Download Table S2, XLSX file, 0.04 MB. Copyright © 2020 Juttukonda et al. 2020 Juttukonda et al. This content is distributed under the terms of the Creative Commons Attribution 4.0 International license.

10.1128/mBio.02555-20.6 TABLE S3 Transcripts significantly* changed# between 100 μM ‘921 and 100 μM ‘921 + 1 mM MnCl_2_ combination treatment. *Significance was defined as FDR < 0.001. #Defined as >2 log_2_ fold change. Download Table S3, XLSX file, 0.03 MB. Copyright © 2020 Juttukonda et al. 2020 Juttukonda et al. This content is distributed under the terms of the Creative Commons Attribution 4.0 International license.

10.1128/mBio.02555-20.7 TABLE S4 Transcripts significantly* changed# by treatment with 1 mM MnCl_2_. *Significance was defined as FDR < 0.001. #Defined as >2 log_2_ fold change. Download Table S4, XLSX file, 0.01 MB. Copyright © 2020 Juttukonda et al. 2020 Juttukonda et al. This content is distributed under the terms of the Creative Commons Attribution 4.0 International license.

**FIG 2:**
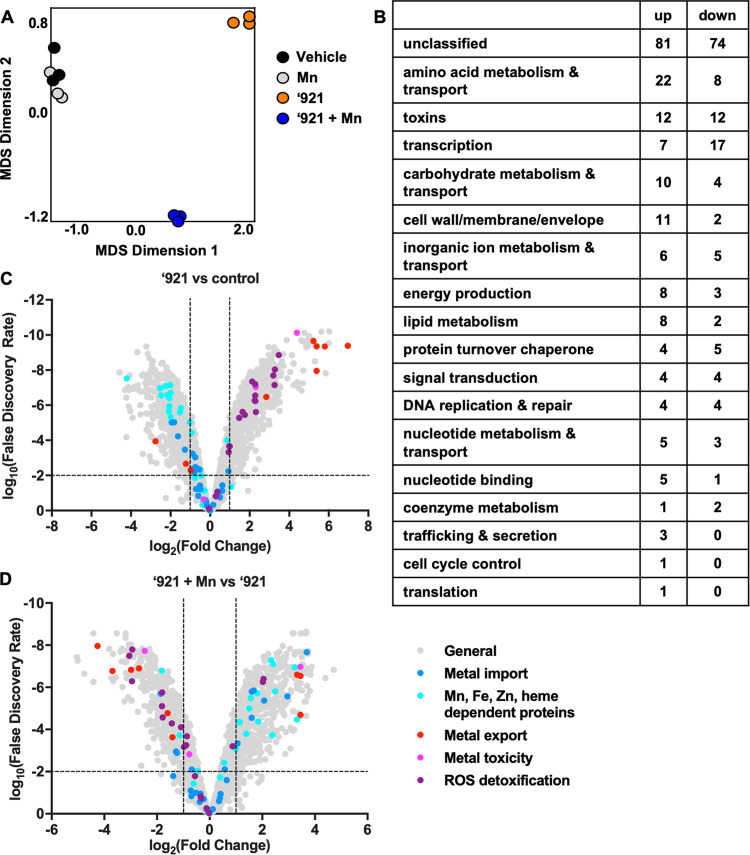
VU0026921 induces a metal toxicity transcriptional response. RNA sequencing was performed on mid-exponential S. aureus Newman cultures exposed to vehicle, 1 mM MnCl_2_, 100 μM ‘921, or 100 μM ‘921 + 1 mM MnCl_2_ for 30 min. (A) Multidimensional scaling visualization of RNA sequencing biological replicates. The *x* and *y* axes are dimensionless units. The closeness of symbols to each other on the 2-dimensional plot represents the relatedness of the transcriptional profiles of the represented data sets. (B) Categories of genes whose transcription was significantly different between 100 μM ‘921 treatment alone and cotreatment with 1 mM MnCl_2_. (C) Fold change (log_2_) of transcript abundance for all S. aureus genes following treatment with VU0026921 compared to vehicle-treated controls. (D) Fold change (log_2_) of transcript abundance for all S. aureus genes following treatment with VU0026921 and 1 mM MnCl_2_ compared to ‘921 alone. For panels C and D, genes involved in metal homeostasis and reactive oxygen species (ROS) detoxification are highlighted. Dashed lines indicate genes with greater than 2-fold change and a false-discovery rate of less than 0.01.

Accordingly, the global transcriptional profiles of vehicle-treated and Mn-treated biological replicates cluster together on a multidimensional scaling (MDS) plot, whereas both of the VU0026921-treated groups cluster independently (Fig. 2A). The COG pathways containing the greatest number of Mn-dependent transcriptional changes between VU0026921 treatment and cotreatment with VU0026921 and Mn are toxin production, amino acid metabolism and transport, and genes involved in transcription (Fig. 2B). This suggests that the addition of Mn does not simply inactivate VU0026921 or reverse VU0026921 activity but instead alters the physiology of the S. aureus cell to promote survival. Similar transcriptional changes, such as upregulation of superantigen-like genes *NWMN_0390-91* and *NWMN_1076-77*, were observed in other unrelated studies and may be a marker of cellular stress (31–33). The global transcriptional profile of S. aureus treated with VU0026921 indicates that the cellular stress induced by this compound causes changes to S. aureus physiology.

We hypothesized that VU0026921 targets Mn uptake or utilization and anticipated that VU0026921 may alter the transcription of genes involved in metal homeostasis. Interestingly, genes encoding transition metal import systems, including Mn, Fe, and Zn import systems, are downregulated or unchanged by VU0026921 treatment (Fig. 2C and Table S5). In contrast, genes encoding exporters for the highly reactive metal Cu and the reactive Fe-containing tetrapyrrole heme are highly upregulated by VU0026921 (Fig. 2C and Table S5). Moreover, genes thought to function in intracellular metal buffering are increased by treatment with VU0026921 (Fig. 2C and Table S5). This transcriptional pattern suggests that VU0026921 induces a metal toxicity response in S. aureus.

10.1128/mBio.02555-20.8 TABLE S5 Expression of genes involved in metal homeostasis and detoxification following treatment with VU0026921 with or without Mn. Download Table S5, XLSX file, 0.01 MB. Copyright © 2020 Juttukonda et al. 2020 Juttukonda et al. This content is distributed under the terms of the Creative Commons Attribution 4.0 International license.

Metals alone and the mismetalation of enzymes can generate ROS, which damage cellular macromolecules (36, 37). Genes involved in detoxification of ROS are upregulated following treatment with VU0026921 (Fig. 2C and Table S5). The transcriptional upregulation of metal export and ROS detoxification systems coupled with unchanged or downregulated genes encoding metal uptake and metal-binding proteins suggests that VU0026921 causes S. aureus to experience metal toxicity. The addition of Mn, which reverses the antimicrobial activity of VU0026921 toward S. aureus, leads to downregulation of metal export genes and ROS-detoxification systems (Fig. 2D). Taken together, these transcriptional changes implicate intracellular metal homeostasis as a target of VU0026921 toxicity.

### VU0026921 causes intracellular metal accumulation in S. aureus.

**FIG 3:**
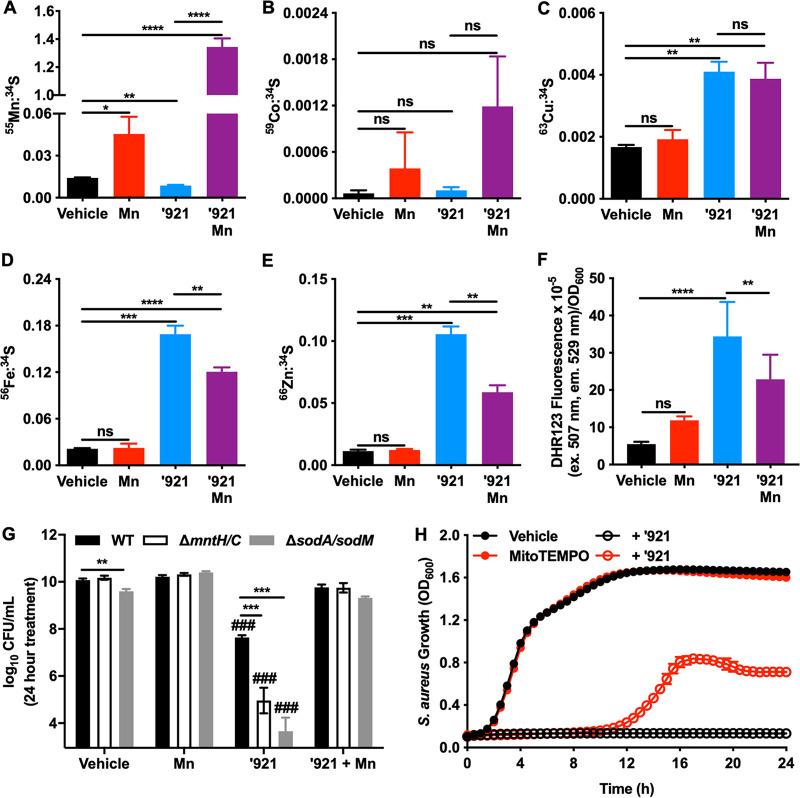
VU0026921 causes metal accumulation and oxidative stress in S. aureus. Metal isotopes ^55^Mn (A), ^59^Co (B), ^63^Cu (C), ^56^Fe (D), and ^66^Zn (E) were measured by ICP-MS in S. aureus Newman treated for 30 min with vehicle, 1 mM MnCl_2_, 100 μM ‘921, or 100 μM ‘921 + 1 mM MnCl_2_. Data are mean ± standard deviation normalized to ^34^S to account for differences in growth of four biological replicates. Statistical significance was determined by one-way ANOVA where ns = *P > *0.05, * = *P < *0.05, ** = *P < *0.01, *** = *P < *0.001, and **** = *P < *0.0001. (F) ROS levels in S. aureus cultures treated with vehicle, 50 μM ‘921, 1 mM MnCl_2_, or 50 μM ‘921 + 1 mM MnCl_2_ for 6 h. Data are mean ± standard deviation for six biological replicates. Statistical significance was determined by one-way ANOVA with Tukey’s multiple-comparison test where ** = *P < *0.01 and **** = *P < *0.0001. (G) CFU recovered following 24-h treatment of mid-exponential-phase cultures of S. aureus Newman, Δ*mntH/C*, or Δ*sodA/sodM* with vehicle, 1 mM MnCl_2_, 100 μM ‘921, or 1 mM Mn + 100 μM ‘921. Data are mean ± standard deviation combined from three independent triplicate experiments, performed on separate days (*n* = 9). Statistical significance was determined by one-way ANOVA with Dunnett’s multiple-comparison test where ### = *P < *0.001 compared to WT vehicle-treated bacteria and ** = *P* < 0.01 and *** = *P < *0.001 for the comparisons indicated by the bars. (H) S. aureus Newman was treated with vehicle, 50 μM ‘921, 80 μM MitoTEMPO, or 50 μM ‘921 + 80 μM MitoTEMPO, and growth was monitored by optical density at 600 nm for 24 h. Data are mean ± standard deviation for six biological replicates.

To determine the impact of VU0026921 treatment on S. aureus metal levels, inductively coupled plasma mass spectrometry (ICP-MS) was performed on S. aureus cells exposed to VU0026921 for 30 min. Cu, Fe, and Zn concentrations increase in cells treated with VU0026921, whereas there is no difference in the concentration of Co, and Mn decreases slightly upon VU0026921 treatment (Fig. 3A to E). This decrease in intracellular Mn may explain why VU0026921 inhibits S. aureus Δ*mntH/C* more than the wild-type strain (Fig. 1B), as this mutant is Mn starved at baseline. In contrast, cells treated with VU0026921 and 1 mM Mn demonstrate significantly higher Mn concentrations than treatment with either compound or Mn alone (Fig. 3A). Additionally, VU0026921- and Mn-cotreated samples have lower Fe and Zn levels than samples treated with compound alone (Fig. 3D and E). Cu levels are not changed when comparing VU0026921 treatment to VU0026921 plus Mn treatment.

Cotreatment with EDTA decreases the intracellular concentrations of Mn, Cu, Fe, and Zn compared to VU0026921 treatment alone, suggesting that EDTA competes with VU0026921 for binding of these metals and further implicating metal uptake as the mechanism of action by VU0026921 (Fig. S2A and C to E). S. aureus is Mn depleted when VU0026921 and EDTA are used as cotreatment (Fig. S2A), explaining why the combination treatment of VU0026921 and EDTA inhibits S. aureus growth so well (Fig. 1E). The combination of VU0026921 and EDTA is more effective at preventing Zn uptake (Fig. S2D) than Fe uptake (Fig. S2E). Presumably, this is due to the formation constant for the EDTA + Zn complex being 158-fold greater than the formation constant for EDTA + Fe (38). Co levels are not affected by VU0026921 or EDTA (Fig. 3B and Fig. S2B). Therefore, VU0026921 alters metal homeostasis and leads to accumulation of Zn, Fe, and Cu in the cell, but Mn accumulation is dependent on excess Mn being present in the medium.

10.1128/mBio.02555-20.2 FIG S2 EDTA lowers ‘921-induced metal uptake, and Co protects S. aureus against VU0026921 killing. (A to E) Metal isotopes ^55^Mn (A), ^59^Co (B), ^63^Cu (C), ^56^Fe (D), and ^66^Zn (E) were measured by ICP-MS in S. aureus Newman treated for 30 min with vehicle, 100 μM EDTA, 100 μM ‘921, or 100 μM ‘921 + 100 μM EDTA. Data are mean ± standard deviation normalized to ^34^S to account for differences in growth of four biological replicates. Statistical significance was determined by one-way ANOVA where ns = *P > *0.05, * = *P < *0.05, ** = *P < *0.01, *** = *P < *0.001, and **** = *P < *0.0001. (F) S. aureus Newman was treated with vehicle or 25 μM ‘921 and increasing molar ratios of CoCl_2_ to ’921. At 6 h following treatment, growth was determined by optical density at 600 nm. Data are mean ± standard deviation for six biological replicates. Statistical significance was determined for each condition compared to vehicle treatment by one-way ANOVA with Tukey’s multiple-comparison test where ns = *P > *0.05 and **** = *P < *0.0001. (G) S. aureus Newman was treated with vehicle, 10 μM ‘921, 100 μM CoCl_2_ or vitamin B_12_, or 10 μM ‘921 + 100 μM CoCl_2_ or vitamin B_12_, and growth was monitored by optical density at 600 nm for 24 h. Data are mean ± standard deviation for six biological replicates. Download FIG S2, TIF file, 2.7 MB. Copyright © 2020 Juttukonda et al. 2020 Juttukonda et al. This content is distributed under the terms of the Creative Commons Attribution 4.0 International license.

Mn is used by S. aureus as both a SOD-dependent and SOD-independent antioxidant; therefore, the hypothesis that VU0026921 induces ROS in S. aureus was tested. VU0026921-induced ROS production was quantified using the compound dihydrorhodamine 123 (DHR123), which is nonfluorescent until it is oxidized by cellular oxidants. VU0026921 induces increased ROS in S. aureus and cotreatment with Mn decreases the amount of ROS generated (Fig. 3F). These data correlate S. aureus ROS levels with VU0026921 killing of S. aureus and rescue of killing by Mn as observed in Fig. 1D.

### Genes involved in Mn import and ROS detoxification protect against VU0026921 toxicity.

Based on the transcriptional profile of S. aureus and the increase in ROS following treatment with VU0026921, we hypothesized that proteins involved in maintaining metal homeostasis may contribute to VU0026921 toxicity by modulating intracellular metal availability. S. aureus strains with mutations in genes related to antioxidant defenses and Mn import were tested for sensitivity to compound VU0026921 in a 24-h killing assay. The deletion of genes encoding the ROS-detoxifying enzymes SodA and SodM enhances susceptibility to VU0026921 in a manner reversed by excess Mn (Fig. 3G). To further test the role of ROS in VU0026921 killing of S. aureus, the superoxide scavenger MitoTEMPO (39) was used to deplete VU0026921-generated ROS. As seen in Fig. 3H, the cotreatment of MitoTEMPO with VU0026921 protects S. aureus from VU0026921 killing but does not completely rescue the phenotype, indicating that VU0026921 does not kill S. aureus solely through ROS generation, which is consistent with Fig. 1E, where VU0026921 can kill S. aureus independently of oxygen. These findings suggest that VU0026921 may act, in part, through generation of superoxide and the resulting downstream oxidants, either through direct action by VU0026921 or through mismetalation that occurs during VU0026921 treatment. Moreover, genetic inactivation of Mn importers MntH and MntC enhances susceptibility to VU0026921, and this susceptibility is reversed by the addition of excess Mn to the growth medium (Fig. 3G). These results suggest that an increased ratio of intracellular Mn relative to other metals protects against VU0026921 toxicity.

### Co and Mn protect S. aureus from VU0026921 killing, while Cu exacerbates killing.

**FIG 4:**
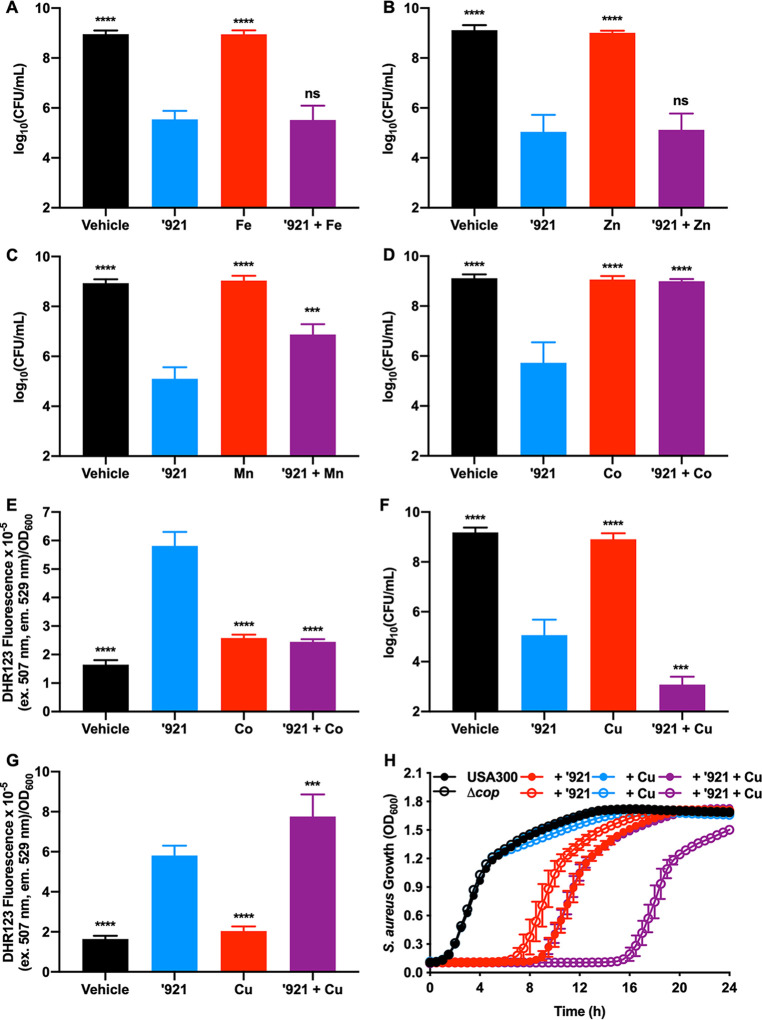
Mn and Co protect S. aureus against VU0026921 toxicity, while Cu exacerbates it. (A to D and F) CFU recovered following 4-h treatment of S. aureus Newman with vehicle, 100 μM ‘921, 100 μM FeSO_4_ (A), ZnCl_2_ (B), MnCl_2_ (C), CoCl_2_ (D), and CuSO_4_ (F), or 10 μM ‘921 + 100 μM metal. Data presented are mean ± standard deviation from triplicate measurements. Statistical significance was determined by one-way ANOVA with Tukey’s multiple-comparison test where ns = *P > *0.05, *** = *P < *0.001, and **** = *P < *0.0001. (E and G) ROS levels in S. aureus cultures treated with vehicle, 50 μM ‘921, 100 μM CoCl_2_ (E) or CuSO_4_ (G), and 50 μM ‘921 + 100 μM metal for 6 h. Data are mean ± standard deviation for six biological replicates. Statistical significance was determined for each condition compared to ‘921 treatment by one-way ANOVA with Tukey’s multiple-comparison test where *** = *P < *0.001 and **** = *P < *0.0001. (H) USA300, an MRSA strain, and USA300 Δ*copAZ* Δ*copBL* (Δ*cop*) were treated with vehicle, 5 μM ‘921, 10 μM CuSO_4_, or 5 μM ‘921 + 10 μM CuSO_4_, and growth was monitored by optical density at 600 nm for 24 h. Data are mean ± standard deviation for six biological replicates.

VU0026921 increases the uptake of transition metals (Fig. 3A to E), so the hypothesis that metals added to the medium will alter the killing of S. aureus by VU0026921 was tested. Neither Fe nor Zn modulates VU0026921 killing (Fig. 4A and B). Consistent with previous experiments, Mn added to the media protects S. aureus from VU0026921 killing (Fig. 4C). Co also protects S. aureus from VU0026921-mediated killing, is more protective than Mn (Fig. 4D), and protects at a much lower molar ratio to VU0026921 than Mn (Fig. S2F). Co protection, similarly to what was seen with Mn, decreases intracellular ROS induced by VU0026921 (Fig. 4E), and the protective effects of Co are not mediated through vitamin B_12_ (Fig. S2G).

In contrast to Co and Mn, cotreatment with Cu exacerbates S. aureus killing by VU0026921 (Fig. 4F), which corresponds with the increase in intracellular Cu observed in VU0026921-treated S. aureus (Fig. 3C). To understand whether Cu export protects against VU0026921 toxicity, we used an MRSA strain of S. aureus with defined Cu export systems. USA300 LAC (40) accounts for most MRSA infections in the United States (41). This S. aureus strain has two systems that protect against Cu toxicity, CopAZ and CopBL (42). Figure 4H demonstrates that VU0026921 is also growth inhibitory to USA300 LAC and that deletion of the Cu export genes (Δ*cop*) increases VU0026921 toxicity in the presence of 10 μM Cu, which is not toxic without VU0026921. The Cu in tryptic soy broth (TSB) alone, approximately 100 nM Cu, is not sufficient to kill the Δ*cop* mutant when treated with VU0026921. These data suggest that VU0026921-facilitated Cu uptake is toxic to S. aureus (Fig. 4F). These data may have implications for future therapies that combine small molecules that induce metal uptake and Cu. Since Cu has strong mismetalation properties based on the Irving-Williams series and can participate in Fenton chemistry, the hypothesis that VU0026921-mediated Cu uptake increases ROS-dependent killing of S. aureus was tested. Cotreatment of Cu with VU0026921 increases ROS compared to VU0026921 treatment alone, correlating with the increased killing seen under these same conditions (Fig. 4G). Taken together, these data confirm that VU0026921 alters S. aureus metal homeostasis and imply that its antimicrobial mechanism relates to the accumulation of intracellular metals and generation of ROS.

### Metal binding stabilizes VU0026921.

**FIG 5:**
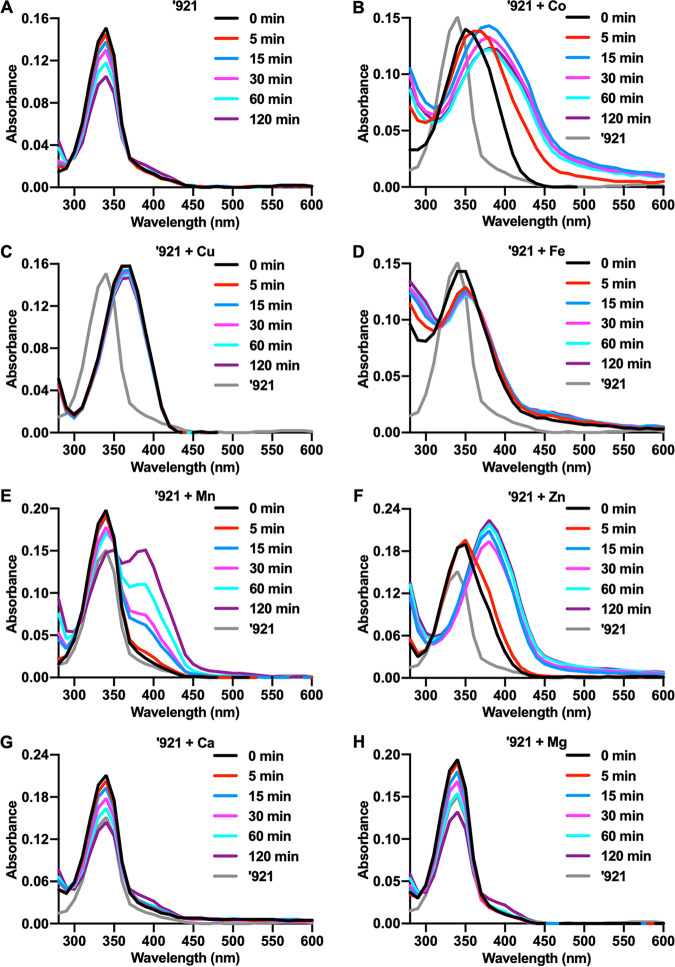
Binding of divalent transition metals stabilized VU0026921. (A to H) Absorbance was measured from 280 nm to 600 nm in 10-nm increments of 0 or 100 μM ‘921 combined with vehicle (A) or 500 μM CoCl_2_ (B), CuSO_4_ (C), FeSO_4_ (D), MnCl_2_ (E), ZnCl_2_ (F), CaCl_2_ (G), or MgCl_2_ (H). Absorbance measurements were taken at 0, 5, 15, 30, 60, or 120 min after the addition of metals. The final absorbance spectra are ‘921 + metal with spectra for metal alone subtracted. Spectra displayed are representative of a single experiment that was performed three times. The spectrum of ‘921 at 0 min was included in each panel as a reference of the compound absorbance alone.

We hypothesized that VU0026921 causes accumulation of cellular metals by directly binding and promoting metal transport into the cell. VU0026921 absorbs light between 300 and 400 nm (Fig. 5A). The absorbance intensity of VU0026921 decreases over time, indicating that the compound is changing or degrading during the 120 min of this experiment (Fig. 5A). The absorbance spectrum of VU0026921 shifts to longer wavelengths in the presence of Co, Cu, Fe, Mn, and Zn (Fig. 5B to F), suggesting that VU0026921 binds these metals. When Cu and VU0026921 are combined, the absorbance shift occurs faster than the plate reader is able to acquire the initial absorbance scan (Fig. 5C). Additionally, the formation constant for the EDTA + Cu complex is over 30,000-fold greater than the formation constant for EDTA + Fe (38), but the decrease in intracellular Cu (Fig. S2C) levels is similar to Fe (Fig. S2D) when comparing VU0026921 + EDTA to VU0026921, further emphasizing that VU0026921 and Cu have rapid and strong binding kinetics (Fig. 5C). The binding kinetics with Mn are considerably slower, and VU0026921 is not completely bound to Mn at the end of the 120-min experiment (Fig. 5E). Slow binding kinetics may explain why so much Mn is needed to protect S. aureus from VU0026921 toxicity. The rapid binding of Cu and the slow binding of Mn with VU0026921 may also explain why treatment with VU0026921 and Mn does not reduce the amount of Cu (Fig. 3C) taken up by the cells as it does for Fe and Zn (Fig. 3D and E). Metals that bind VU0026921 appear to stabilize the molecule, so that the decrease in absorbance intensity seen in VU0026921 alone is decreased. The divalent cations Ca and Mg, which do not appear to bind VU0026921, also do not have the stabilizing effects on the molecule seen for the other metals (Fig. 5G and H). The binding of VU0026921 to these metals as well as the increase in intracellular metal concentrations (Fig. 3A to E) supports the hypothesis that VU0026921 promotes metal transport into the cell.

### Preliminary structure-activity relationship study of VU0026921 reveals functional groups required for toxicity.

**FIG 6:**
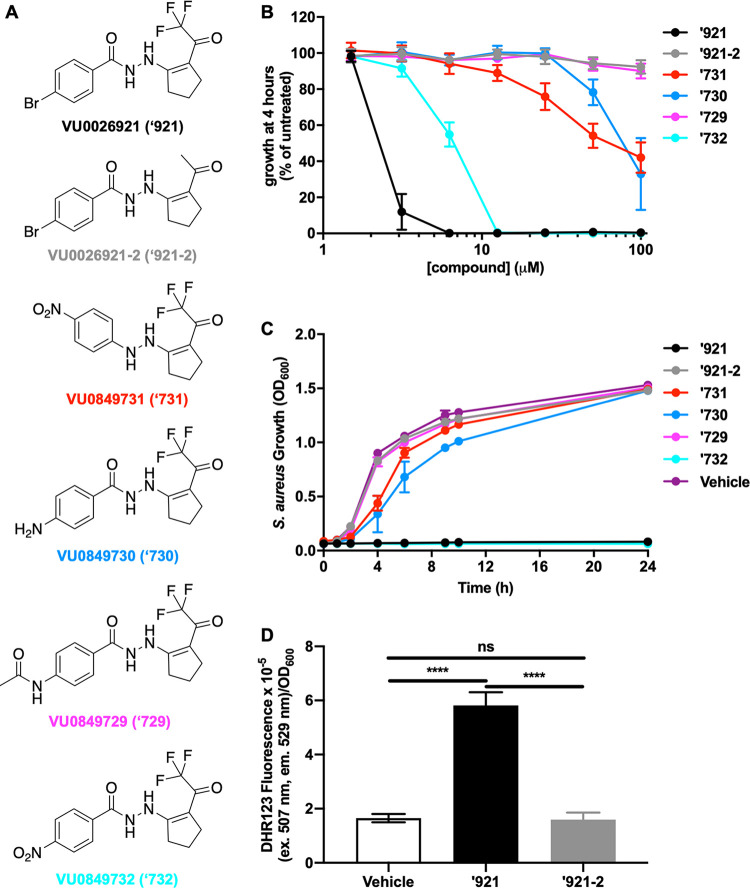
Chemical features of VU0026921 required for toxicity. (A) Chemical structures of VU0026921 and analogs. (B) Relative potency of 100 μM (each) analog—VU0026921 (‘921), VU0849731 (‘731), VU0849732 (‘732), VU0849730 (‘730), or VU0849729 (‘729)—measured as growth of S. aureus as a percentage of untreated cells at 4 h for each compound tested at the indicated concentrations. Data are mean ± standard deviation from triplicate measurements. (C) S. aureus Newman was treated with vehicle, 100 μM ‘921, or 100 μM ‘921 analog, and growth was monitored by optical density at 600 nm for 24 h. Data are mean ± standard deviation from triplicate measurements. (D) ROS levels in S. aureus cultures treated with vehicle, 50 μM ‘921, or 50 μM ‘921-2 for 6 h. Data are mean ± standard deviation for six biological replicates. Statistical significance was determined by one-way ANOVA with Tukey’s multiple-comparison test where ns = *P > *0.05 and **** = *P < *0.0001.

Five small molecules were synthesized based on the VU0026921 backbone to determine what structural features are required to support metal-dependent killing activity and to identify where the molecule can be modified with affinity tags for target identification and future probe improvement (Fig. 6A). Additionally, VU0026921 is not stable in TSB (Fig. 5A and Fig. S1B and C), so an active compound with more medium stability will aid in future studies of small-molecule-mediated metal uptake. Trifluoromethyl ketones readily hydrate and serve as Zn-binding hydroxamic acid replacements in histone deacetylase (HDAC) inhibitors (43); therefore, we first assessed a methyl ketone replacement for the trifluoromethyl group of VU0026921 (VU0026921-2). As anticipated, methyl ketone VU0026921 does not inhibit S. aureus growth (Fig. 6B and C). Additionally, the increase in ROS observed following the treatment of S. aureus with VU0026921 does not occur in cells treated with VU0026921-2, correlating ROS levels with the bactericidal ability of these compounds in S. aureus (Fig. 6D). Likewise, substitution of the acyl hydrazide linker for a hydrazine linker (VU0849731) decreases the antimicrobial activity (Fig. 6B and C), suggesting that metal binding and/or NH acidity may be important for antimicrobial activity. The importance of the latter property is further supported by the finding that replacement of the para-bromo group with less electron-withdrawing groups such as para-amino (VU0849730) or N-acetamido (VU0849729) results in loss of activity (Fig. 6B and C). In sharp contrast, replacement of the bromo group with an electron-withdrawing para-nitro group (VU0849732) only mildly impacts antimicrobial activity (Fig. 6B and C). Together, these data suggest that electron-withdrawing trifluoromethyl ketone and acyl hydrazide groups across the compound contribute to activity.

### VU0026921 does not inhibit fatty acid biosynthesis.

**FIG 7:**
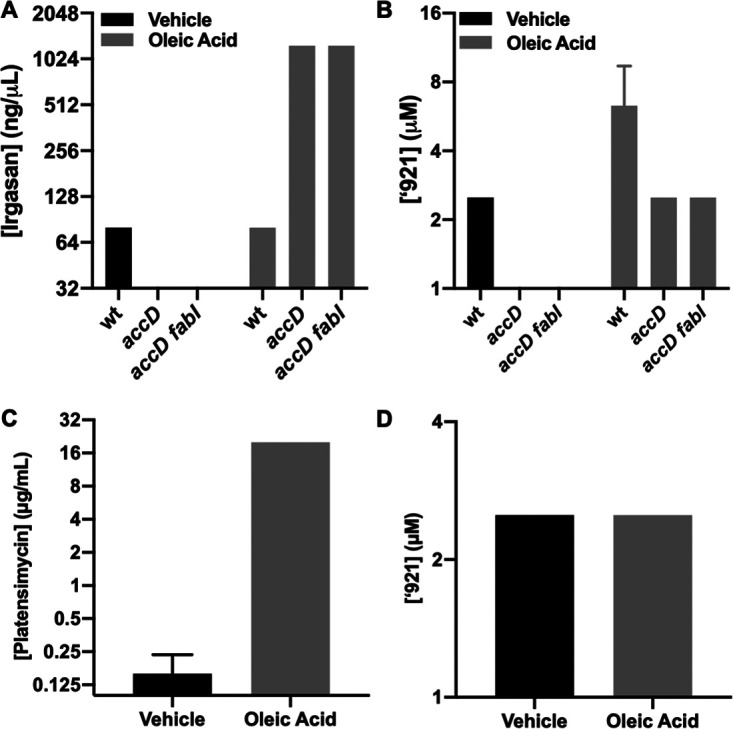
VU0026921 S. aureus killing is not through fatty acid biosynthesis inhibition. (A and B) MIC of irgasan (A), a FabI inhibitor that halts fatty acid biosynthesis and inhibits S. aureus strain RN4220, and VU0026921 (B). Irgasan has an increased MIC in S. aureus RN4220 mutants with fatty acid biosynthesis disabled when cotreated with oleic acid, while ‘921 (B) does not, indicating that ‘921 does not inhibit fatty acid biosynthesis. Note: no growth is observed for *accD* mutants in the absence of oleic acid. (C and D) Platensimycin (C), a FabF inhibitor, has an increased MIC in E. faecalis when cotreated with oleic acid, while ‘921 (D) does not, suggesting that ‘921 does not inhibit fatty acid biosynthesis in E. faecalis. All data are mean ± standard deviation for triplicate measurements. Note: 10 μg/ml was the highest concentration of platensimycin tested. The actual MIC of platensimycin against E. faecalis in the presence of oleic acid may be higher.

Due to structural similarities between VU0026921 and isoniazid, the hypothesis that the VU0026921 mechanism of action is through the inhibition of fatty acid biosynthesis was tested. Irgasan, a FabI inhibitor, was used as a positive-control compound in these studies. In wild-type S. aureus, inhibition of fatty acid biosynthesis is not reversible by the addition of external fatty acids (44). However, a mutant in acetyl coenzyme A carboxylase *accD* both is a fatty acid auxotroph and is resistant to inhibitors of fatty acid biosynthesis (45) (Fig. 7A). This increase in MIC is not observed in *accD* mutants when VU0026921 is cotreated with oleic acid (Fig. 7B), indicating that VU0026921 does not inhibit S. aureus through the inhibition of fatty acid biosynthesis. In contrast to S. aureus, fatty acid biosynthesis inhibition in wild-type *Lactobacillales* is reversible with fatty acids (45). Addition of oleic acid reversed the activity of a FabF inhibitor, platensimycin, in Enterococcus faecalis, increasing its MIC by at least 100-fold (Fig. 7C). However, no MIC increase is observed when oleic acid is added in combination with VU0026921 (Fig. 7D), indicating that VU0026921 does not inhibit fatty acid biosynthesis in E. faecalis. In total these data demonstrate that VU0026921, despite having structural similarities to isoniazid, does not target the same cellular process as isoniazid.

### VU0026921 kills Gram-positive bacteria.

**FIG 8:**
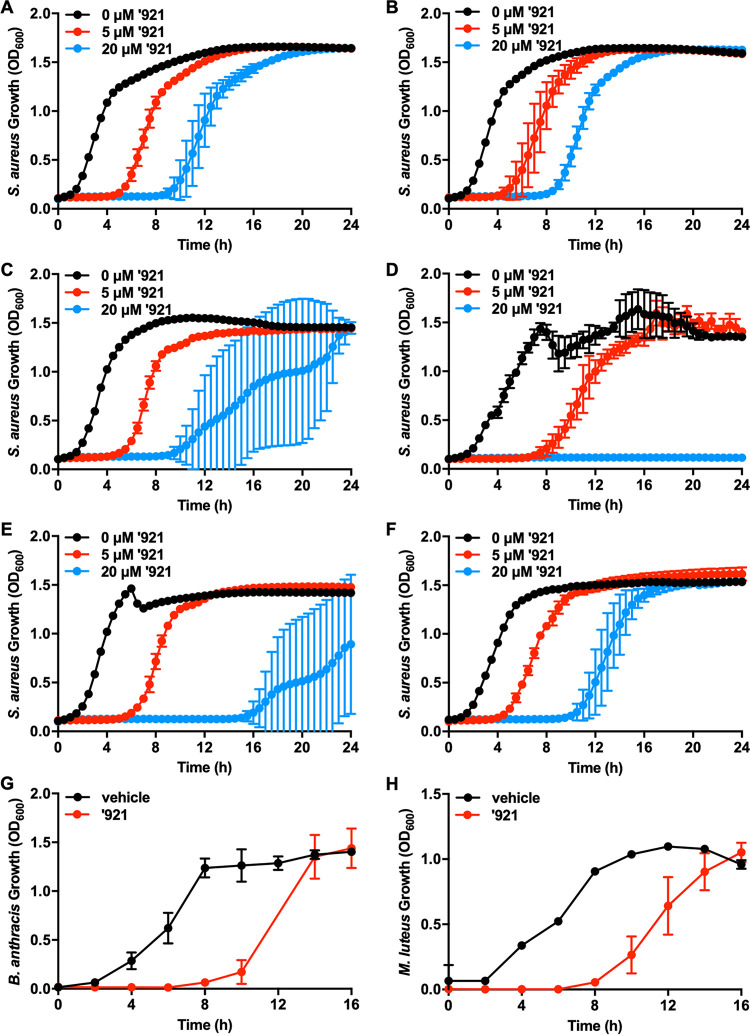
VU0026921 is growth inhibitory toward Gram-positive bacteria. (A to F) S. aureus MW2 (A), UAMS1 (B), HG003 (C), 8325-4 (D), RN6390 (E), and SH1000 (F) were treated with vehicle or 5 μM or 20 μM ‘921, and growth was monitored by optical density at 600 nm for 24 h. Data are mean ± standard deviation from triplicate measurements. (G and H) The Gram-positive organisms Bacillus anthracis (G) and Micrococcus luteus (H) were treated with vehicle or 5 μM ‘921, and growth was monitored by optical density at 600 nm for 16 h. Data are mean ± standard deviation from quadruplicate measurements.

As proof-of-concept that the process of generating metal toxicity has potential as an antibiotic strategy, we assessed the antimicrobial activity of VU0026921 across a range of bacterial pathogens. In addition to inhibiting the growth of methicillin-sensitive S. aureus (Fig. 1), VU0026921 inhibits the growth of MRSA (Fig. 4H) as well as S. aureus strains MW2 (Fig. 8A), UAMS1 (Fig. 8B), HG003 (Fig. 8C), 8325-4 (Fig. 8D), RN6390 (Fig. 8E), and SH1000 (Fig. 8F). VU0026921 also inhibits the growth of the Gram-positive organisms E. faecalis (Fig. 7D), Bacillus anthracis (Fig. 8G), and Micrococcus luteus (Fig. 8H). B. anthracis is a highly virulent organism with potential for use as a bioweapon (46) and a rapidly increasing infection rate resulting from intravenous, illicit drug use (47). M. luteus is often used to screen compounds for antimicrobial activity (48, 49). VU0026921 does not inhibit the growth of the Gram-negative species Escherichia coli DH5α (Fig. S3A), E. coli MG1655 (Fig. S3C), Pseudomonas aeruginosa PAO1 (Fig. S3E), P. aeruginosa PA14 (Fig. S3F), and Klebsiella pneumoniae TOP2 (Fig. S3G). The Gram-negative species E. coli SLO1B (Fig. S3B), E. coli BL21 (Fig. S3D), and Acinetobacter baumannii 17978 (Fig. S3H) are all slightly inhibited by VU0026921, although at concentrations more than 10-fold higher than what is needed to inhibit growth of Gram-positive species. Together, these data suggest that VU0026921 is far more antimicrobial against Gram-positive than Gram-negative organisms. These results provide preliminary evidence that small molecules can be developed that target metal toxicity in Gram-positive pathogens.

10.1128/mBio.02555-20.3 FIG S3 VU0026921 is not growth inhibitory towards Gram-negative bacteria. The Gram-negative organisms E. coli DH5α (A), E. coli SLO1B (B), E. coli MG1655 (C), E. coli BL21 (D), P. aeruginosa PAO1 (E), P. aeruginosa PA14 (F), K. pneumoniae TOP2 (G), and A. baumannii 17978 (H) were treated with vehicle or 0 μM (untreated) or 160 μM ‘921, and growth was monitored by optical density at 600 nm for 24 h. Data are mean ± standard deviation from triplicate measurements. Download FIG S3, TIF file, 2.7 MB. Copyright © 2020 Juttukonda et al. 2020 Juttukonda et al. This content is distributed under the terms of the Creative Commons Attribution 4.0 International license.

## DISCUSSION

There is significant need to develop antimicrobials to combat drug-resistant bacteria. Here, we demonstrate that intracellular metal homeostasis can be targeted by small molecules and that perturbation of metal homeostasis by small molecules has antimicrobial effects. We propose targeting metal homeostasis as it represents an avenue toward additional novel antibiotic discovery. We identify VU0026921 as a small molecule toxic to S. aureus and present evidence that VU0026921 disrupts metal homeostasis. Divalent cations modulate the antimicrobial activity and stability in solution of VU0026921. Exposure of S. aureus to VU0026921 upregulates metal and ROS detoxification systems, and ROS levels correlate with VU0026921 toxicity. S. aureus accumulates metals following incubation with VU0026921. Finally, inactivation of genes involved in Mn import and utilization enhances S. aureus susceptibility to VU0026921. VU0026921 has antimicrobial activity against all S. aureus strains tested as well as other Gram-positive organisms. Together, these results establish VU0026921 as a probe for studying Gram-positive microbial metal metabolism and support the possibility of developing small-molecule antimicrobials that target metal uptake.

VU0026921 exerts its antimicrobial activity by targeting metal import and homeostasis rather than by acting as an extracellular metal chelator and sequestering metals from S. aureus. While VU0026921 binds to divalent transition metals and inhibits bacterial growth in a manner both reversed and exacerbated by the addition of different metals, properties that would be expected for a metal chelator, it does so in a nonstoichiometric manner. Furthermore, an extracellular metal chelator would be expected to inhibit Gram-negative as well as Gram-positive bacteria, but VU0026921 slightly inhibits the growth of some Gram-negative organisms at concentrations more than 10-fold higher than what is needed to inhibit Gram-positive organisms. This result suggests that penetration into the cell or a specific cellular target is required for the antimicrobial activity of VU0026921. Finally, ICP-MS results demonstrate that VU0026921 increases cellular metal levels in S. aureus, whereas an extracellular chelator would be expected to decrease cellular metal levels.

While the exact mechanism of action for VU0026921 remains unknown, we propose a model of activity wherein VU0026921 causes an influx of metal, leading to metal-dependent toxicity. This model is supported by the increase in cellular metal levels following treatment with VU0026921. VU0026921 may potentiate metal influx by interacting with metal transporters, such as by locking these proteins into an open conformation. A second possibility is that VU0026921 is a metallophore that binds metals in growth medium and facilitates transport of these metals into S. aureus. Both of these models are consistent with the observation that metals present in growth medium become concentrated in the S. aureus cell following treatment with VU0026921. Notably, Mn does not accumulate when cells are treated with VU0026921 in TSB alone, potentially due to the slow kinetics of binding between VU0026921 and Mn compared to other metals tested. The proposed model of VU0026921-mediated metal influx is also supported by transcriptomic analyses, which reveal that treatment with compound leads to upregulation of metal efflux genes and downregulation of genes encoding metal-binding proteins, the transcriptional pattern expected to restore metal equilibrium. The additional transcriptional changes observed in the RNA sequencing results are most likely secondary effects of the stress experienced by the cell following dramatic changes in metal levels. We were unable to identify a nonessential gene required for VU0026921 toxicity in S. aureus or isolate a VU0026921-resistant S. aureus strain. These findings suggest that VU0026921 has multiple targets within the cell or has an antimicrobial activity that does not require a specific cellular target, such as generation of ROS, which directly correlates with killing by VU0026921 and metals.

The question of how intracellular metal accumulation causes cellular toxicity in VU0026921-treated cells remains unanswered. While this study does not elucidate the cellular targets of metal-dependent toxicity, one possible mechanism is metal catalyzing the production of ROS, such as through the Fenton and Haber-Weiss reactions (50). Deletion of *sodA* and *sodM* enhanced VU0026921 toxicity, and cotreatment with MitoTEMPO, a superoxide dismutase mimetic, is partially protective, implicating ROS in VU0026921 toxicity. One metal that can be toxic to cells through all of the above mechanisms is Cu. VU0026921 treatment induces Cu uptake by S. aureus and increased cellular ROS. Inactivation of the S. aureus Cu detoxification systems exacerbates the toxicity of VU0026921 cotreated with nontoxic Cu levels, indicating a mechanism of Cu toxicity that proceeds though the accumulation of intracellular Cu.

Mn rescues VU0026921 aerobically, and Mn has been previously described as an antioxidant and may protect S. aureus from ROS by forming antioxidant small molecules (26, 51, 52), serving as a cofactor for superoxide dismutase (25, 28), or replacing oxidized Fe in mononuclear enzymes (53). Mn does not protect S. aureus from anaerobic VU0026921 toxicity, demonstrating that Mn protects S. aureus from oxygen-dependent toxicity, such as ROS. A second possible mechanism of metal toxicity is replacement of cognate metals in metalloenzymes (9, 54, 55). This mechanism may explain VU0026921-mediated toxicity in anaerobically grown cells and why antioxidants do not completely protect S. aureus. Cotreatment with Mn and VU0026921 causes massive accumulation of Mn within the S. aureus cell, suggesting that Mn may protect S. aureus from VU0026921 toxicity by blocking the import of other, more toxic metals. This is further supported by the decrease in cellular Fe and Zn levels in S. aureus cotreated with VU0026921 and Mn compared to cells treated with VU0026921 alone. Exogenous Co protects S. aureus from VU0026921 killing but is not accumulated under any of the conditions tested by ICP-MS even though Co is present in TSB at similar levels as Cu. Cellular Cu levels, which are increased upon VU0026921 treatment, do not decrease like Fe and Zn when 1 mM Mn is cotreated with VU0026921. The addition of exogenous Mn represents 10,000-fold more Mn than Cu in the medium, which is insufficient to compete with Cu uptake, which correlates with the rapid binding kinetics of Cu and slow binding kinetics of Mn with VU0026921.

Co, in contrast to Mn, completely protects S. aureus from VU0026921 killing through a mechanism that eliminates VU0026921-induced cellular ROS and is independent of vitamin B_12_, the major Co-containing molecule in cells. Co also maximally protects S. aureus from VU0026921 killing at much lower metal/VU0026921 molar ratios. A Co/VU0026921 molar ratio of 2 gives complete protection, while it takes an Mn/VU0026921 ratio of 50 to get 80% rescue. The near-unity stoichiometry of protection by Co is consistent with Co binding to VU0026921, preventing other metals from binding. However, further experiments are necessary to completely understand the role of Co in protecting S. aureus from VU0026921 killing.

Recent studies emphasizing the importance of metal homeostasis in bacterial pathogenesis have garnered enthusiasm for developing metal-dependent antimicrobials (17–22). Most previous studies emphasized the development of antimicrobials to restrict metal acquisition by pathogens rather than antimicrobials that cause metal accumulation and toxicity. However, bacterial pathogens also must balance metal toxicity during infection. Cefiderocol, a siderophore cephalosporin that increases cellular Fe levels in bacteria, was recently approved for the treatment of Gram-negative urinary tract infections, emphasizing that metal uptake can be exploited for antimicrobial development (56). There are tissue-specific fitness defects for S. aureus and Streptococcus pneumoniae strains lacking an Mn efflux system (34, 57); a Helicobacter pylori strain lacking a Cu, Cd, and Ni efflux pump (58); and several bacterial pathogens lacking Cu detoxification systems (59). An intriguing possibility raised by this study is the concept of targeting specific infectious niches where metal toxicity occurs. However, several key challenges will have to be overcome for VU0026921 derivatives to be used as effective antimicrobials. First, VU0026921 appears to be unstable in aqueous medium and rapidly loses activity in solution, and future compound development will require identifying active analogs with improved stability. Another caveat is that VU0026921 increases Mn levels in mammalian cells in the presence of high extracellular Mn levels (23), and any clinical studies must closely investigate the potential for causing deleterious side effects in the host. Therefore, VU0026921 is best suited as a chemical probe used to expand our understanding of Gram-positive metal homeostasis, and this initial work with VU0026921 provides proof-of-concept evidence that metal toxicity can be exploited as an antimicrobial strategy against Gram-positive pathogens through the activity of a small molecule.

## MATERIALS AND METHODS

### Bacterial strains and reagents.

The strains used in this study are described in Table S6 in the supplemental material. S. aureus strains were grown in tryptic soy broth (TSB); M. luteus, E. coli, P. aeruginosa, A. baumannii, and K. pneumoniae strains were grown in lysogeny broth (LB); and B. anthracis was grown in brain heart infusion broth (BHI). All cultures were grown aerobically at 37°C with 180-rpm shaking. Solid medium contained 1.5% agar. For all assays, bacterial strains were streaked from −80°C stocks 2 days prior to the assay. Overnight cultures were grown for 12 to 15 h in 5 ml medium in 15-ml conical tubes.

10.1128/mBio.02555-20.9 TABLE S6 Bacterial strains used in this study. Download Table S6, XLSX file, 0.01 MB. Copyright © 2020 Juttukonda et al. 2020 Juttukonda et al. This content is distributed under the terms of the Creative Commons Attribution 4.0 International license.

### Creation of USA300 Δ*copAZ* Δ*copBL*.

JMB1100 chromosomal DNA was used as a template for PCRs. Escherichia coli PX5α (Protein Express) was used as a cloning host and for plasmid propagation. Plasmids were isolated and transformed into S. aureus strain RN4220 (60) using a standard protocol (61), and assays were conducted using phage 80α (62). All strains were verified by PCR and/or sequencing.

Construction of plasmids for mutant generation was done using yeast homologous recombination cloning as previously outlined (63, 64). The following primer pairs were used to create the amplicons necessary to make pJB38_Δ*copAZ*: Ycc Pjb38 for and YCC CopAZ rev; CopAZ Up for and CopAZ up rev; CopAZ dwn for and pJB38 CopAZ rev. The amplified PCR fragments were combined with EcoRI-linearized pJB38 plasmid and transformed into Saccharomyces cerevisiae FY2. Mutant strains were constructed using the pJB38 allelic exchange as described previously (65). The Δ*copAZ* Δ*copBL* strain (*cop*-) was created using the Δ*copBL* mutant (JMB7901) (ΔSAUSA300_0078-0079) (42) and pJB38_Δ*copAZ*.

### Compound acquisition and storage.

The compounds in the 39-compound toolbox were purchased from Chemical Diversity (San Diego, CA) and stored as 10 mM stocks in dimethyl sulfoxide (DMSO) at −80°C. Compound VU0026921 was repurchased three times and confirmed to exert similar activity in each batch. Additionally, the structural integrity of purchased VU0026921 was confirmed by nuclear magnetic resonance (NMR). Finally, VU0026921 and VU0026921 analogs were resynthesized by the Vanderbilt Chemical Synthesis Core and used to confirm antimicrobial activity and Mn rescue.

### Compound preparation.

**(i) 4-Bromo-*N*′-(2-(2,2,2-trifluoroacetyl)cyclopent-1-en-1-yl)benzohydrazide (VU0026921).** To a solution of 2-(2,2,2-trifluoroacetyl)cyclopentan-1-one (0.562 ml, 4.65 mmol) in ethanol (10 ml) was added a solution of 4-bromobenzohydrazide (1.0 g, 4.65 mmol) in ethanol (12 ml) dropwise. The reaction mixture was stirred at room temperature for 1 h, solvent was removed *in vacuo*, and the residue was recrystallized (ethanol) to afford 792 mg (74%) of the derived hydrazone as a white solid: ^1^H NMR (deuterated methanol [MeOD], 400 MHz) δ (ppm) 7.71 (d, *J *= 8.4 Hz, 2H), 7.59 (d, *J *= 8.4 Hz, 2H), 2.79 (t, *J *= 7.2 Hz, 2H), 2.70 (t, *J *= 7.6 Hz, 2H), 1.92 (t, *J *= 7.2 Hz, 2H); liquid chromatography-mass spectrometry (LC-MS) (electrospray ionization [ESI]) tR: 1.09 min (>99%, evaporative light scattering detection [ELSD]), *m/z*: 377.0 [M+H]^+^.

**(ii) 2,2,2-Trifluoro-1-(2-(2-(4-nitrophenyl)hydrazinyl)cyclopent-1-en-1-yl)ethan-1-one (VU0849731).** ^1^H NMR (MeOD, 400 MHz) δ (ppm) 8.19 (d, *J *= 9.2 Hz, 2H), 6.96 (d, *J *= 9.2 Hz, 2H), 2.82 to 2.72 (m, 4H), 2.04 to 1.94 (m, 2H).

**(iii) 4-Nitro-*N*′-(2-(2,2,2-trifluoroacetyl)cyclopent-1-en-1-yl)benzohydrazide (VU0849732).** ^1^H NMR (MeOD, 400 MHz) δ (ppm) 8.38 (d, *J *= 8.8 Hz, 2H), 8.11 (d, *J *= 8.8 Hz, 2H), 2.85 to 2.78 (m, 4H), 1.99 (q, *J *= 7.6 Hz, 2H); LC-MS (ESI) tR: 1.01 min (>99%, ELSD), *m/z*: 344.0 [M+H]^+^.

**(iv) 4-Amino-*N*′-(2-(2,2,2-trifluoroacetyl)cyclopent-1-en-1-yl)benzohydrazide (VU0849730).** To a solution of 4-nitro-*N*′-(2-(2,2,2-trifluoroacetyl)cyclopent-1-en-1-yl)benzohydrazide (50 mg, 0.15 mmol) in ethyl acetate (EtOAc), followed by tin chloride (142 mg, 0.75 mmol). After stirring for 4 h at 80°C, reaction mixture was poured into NaOH solution (2 ml, 2 M), yellow product was recrystallized from methanol (MeOH) (33 mg, 70%). ^1^H NMR (DMSO, 400 MHz) δ (ppm) 7.67 (d, *J *= 8.8 Hz, 2H), 6.53 (d, *J *= 8.8 Hz, 2H), 5.87 (s, -NH_2_), 2.81 to 2.76 (m, 2H), 1.99 to 1.91 (m, 2H), 1.28 to 1.11 (m, 2H); LC-MS (ESI) tR: 0.83 min (>99%, ELSD), *m/z*: 314.1 [M+H]^+^.

**(v) *N*-(4-(2-(2-(2,2,2-Trifluoroacetyl)cyclopent-1-en-1-yl)hydrazine-1-carbonyl)phenyl) acetamide (VU0849729).** To a solution of 4-amino-*N*′-(2-(2,2,2-trifluoroacetyl)cyclopent-1-en-1-yl)benzohydrazide (55 mg, 0.176 mmol) at 0°C in dichloromethane (10 ml) was added acetic anhydride (33 μl, 0.35 mmol). The reaction mixture was stirred at room temperature for 16 h, quenched with saturated NaHCO_3_, and extracted with dichloromethane (3 × 20 ml). Organic extracts were combined, dried over MgSO_4_, and concentrated *in vacuo*. The residue was purified by column chromatography using dichloromethane-methanol (Combi-flash Rf, 0% to 10% MeOH gradient) to afford yellow solid (44 mg, 72%). ^1^H NMR (DMSO, 400 MHz) δ (ppm) 10.22 (s, -NH), 7.94 (d, *J *= 8.8 Hz, 2H), 7.67 (d, *J *= 8.8 Hz, 2H), 2.85 to 2.80 (m, 2H), 2.60 to 2.45 (m, 2H), 2.08 (s, 3H), 2.01 (t, *J *= 8.0 Hz, 2H); LC-MS (ESI) tR: 0.87 min (>99%, ELSD), *m/z*: 356.1 [M+H]^+^.

### Antimicrobial growth assays.

All growth curves were carried out in the rich growth medium described for each strain above. For each growth curve, bacterial strains were streaked from −80°C stocks 2 days prior to the assay. Overnight cultures were treated at stationary phase or grown for 12 to 15 h in 5 ml of medium in 15-ml conical tubes, and 1 μl of overnight culture was added to 100 μl of medium in a round-bottom 96-well plate (Corning, Corning, NY). MnCl_2_ (Thermo Fisher, Waltham, MA, or Sigma Millipore, St. Louis, MO) was prepared as a 100 mM stock in deionized water, sterile filtered, and stored at room temperature. Individual components of the growth assay were added to the 96-well plate in the following order: growth medium, growth medium containing additional Mn, and growth medium containing compound. Immediately after adding compound, cultures were added and optical density absorbance at 600 nm (OD_600_) at the zero-time point was established. Ninety-six-well plates were grown at 37°C in a shaking incubator and removed periodically for OD_600_ measurements with a plate reader (BioTek, Winooski, VT). Alternatively, kinetic reads were carried out in the plate reader at 37°C with linear shaking.

### VU0026921 compound stability in medium.

VU0026921 was diluted to 50 μM or 3 μM in TSB and incubated shaking at 37°C for 0, 4, 8, or 24 h. After incubation in TSB, kinetic growth curves were performed in S. aureus Newman as described above.

### Growth curve VU0026921 killing assays.

Overnight cultures of S. aureus strain Newman were diluted 1:100 into 96-well plates containing TSB, MnCl_2_, and VU0026921 at the indicated concentrations and grown at 37°C in a shaking incubator. At 0, 2, 4, 6, and 8 h postinoculation, 5 μl of culture was removed, serially diluted in sterile phosphate-buffered saline (PBS; Corning, Corning, NY), and spot plated onto tryptic soy agar (TSA). CFU were calculated by dilution factor per 1 ml of culture.

### Bacterial killing assays.

Overnight cultures of S. aureus were treated at stationary phase or diluted 1:100 into 5 ml TSB in 15-ml conical tubes and incubated at 37°C in a shaking incubator until cultures reached OD_600_ of 0.4. Mid-exponential cultures were pelleted at 3,220 × *g* for 10 min, and pellets were resuspended in 2.5 ml TSB. TSB, 25 μl of resuspended culture, MnCl_2_, and VU0026921 were added to a final volume of 100 μl and final concentrations of 1 mM for MnCl_2_ and 100 μM for ‘921. For Fig. 1E, EDTA (Millipore Sigma, St. Louis, MO) was added to a final concentration of 0 or 1 mM. For Fig. 4A to E, MnCl_2_, ZnCl_2_ (Millipore Sigma, St. Louis, MO), CoCl_2_ (Thermo Fisher, Waltham, MA), CuSO_4_ (Millipore Sigma, St. Louis, MO), or FeSO_4_ (Millipore Sigma, St. Louis, MO) was added to a final concentration of 100 μM. After incubation at 37°C for the indicated lengths of time, 10 μl of culture was removed, serially diluted in sterile PBS, and spot plated onto TSA for CFU enumeration.

### Anaerobic growth curves and killing assays.

For experiments performed under anaerobic conditions, an anaerobic chamber was employed (Coy Laboratory Products, Grass Lake, MI). S. aureus Newman WT was streaked onto TSA and grown aerobically for 24 h at 37°C. Cultures were started from single colonies in 5 ml of anaerobic TSB and grown at 37°C for 15 h. For growth curves, overnight cultures were subcultured 1:100 into 100 μl of anaerobic TSB containing 0 or 1 mM MnCl_2_ and 0, 1.25, 5, 10, or 20 μM VU0026921 in a round-bottom 96-well plate (Corning, Corning, NY) covered with a Breathe-Easy gas-permeable seal (Sigma, St. Louis, MO). Growth was measured by optical density over time in a BioTek Synergy H1. For killing assays, overnight cultures were diluted 1:100 into 100 μl anaerobic TSB in a round-bottom 96-well plate and incubated at 37°C with shaking until cultures reached OD_600_ of 0.35. Sterile water or MnCl_2_ to a final concentration of 100 μM or 1 mM was added as well as sterile DMSO or VU0026921 to a final concentration of 100 μM. After anaerobic incubation at 37°C with shaking for 24 h, 5 μl of culture was removed, serially diluted in sterile PBS, and spot plated onto TSA for CFU enumeration.

### Growth for transcriptome sequencing and RNA isolation.

Overnight cultures of S. aureus strain Newman were diluted 1:100 into 5 ml TSB in 15-ml conical tubes and grown to mid-exponential phase (OD_600_ = 0.4). Cells were pelleted at 3,220 × *g* for 10 min at room temperature, supernatants were discarded, and cells were resuspended in 2.5 ml TSB. TSB, VU0026921 or vehicle control, Mn or vehicle control, and 2 ml of resuspended bacterial culture were added to a 15-ml conical tube to a final volume of 8 ml and final concentration of 0 or 100 μM VU0026921 and 0 or 1 mM MnCl_2_. Following incubation at 37°C with 180-rpm shaking for 30 min, 10-μl aliquots were removed for CFU plating and cells were harvested by centrifugation at 6,000 × *g* for 10 min at 4°C. Pellets were resuspended in LETS buffer (0.1 M LiCl, 10 mM EDTA, 10 mM Tris HCl, pH 7.4, 1% SDS), homogenized in a bead beater (Fastprep-24; MP Biomedical, Santa Ana, CA) with Lysing Matrix B beads (MP Biomedical, Santa Ana, CA) at a speed of 6 m/s for 45 s, heated at 55°C for 5 min, and centrifuged for 10 min at 15,000 rpm. The upper phase was collected, mixed with 1 ml TRI Reagent (Sigma, St. Louis, MO), and incubated for 5 min at room temperature. Chloroform (0.2 ml, Acros Organics, Waltham, MA) was added, samples were vigorously shaken for 15 s, and samples were incubated at room temperature for 2 min. Following centrifugation at 4°C for 15 min, 600 μl of the upper aqueous phase was collected, and RNA was precipitated with 1 ml isopropanol (Sigma, St. Louis, MO). RNA was washed with 70% ethanol (Sigma, St. Louis, MO) and resuspended in 100 μl DNase-free, RNase-free water (Thermo Fisher, Waltham, MA). DNA contamination was removed by treatment with 8 μl RQ1 enzyme (Promega, Madison, WI), 12 μl 10× RQ1 buffer, and 2 μl RiboLock RNase inhibitor (Thermo Fisher, Waltham, MA) for 2 h at 37°C. DNase was removed, samples were further purified by the RNeasy kit (Qiagen, Hilden, Germany) using the manufacturer’s instructions, and RNA was stored long-term at −80°C.

### RNA sequencing.

RNA sequencing was performed by Vanderbilt Technologies for Advanced Genomics Core Facility (VANTAGE). RNA sequencing was performed on three biological replicates per condition. Total RNA quality was assessed using the 2100 Bioanalyzer (Agilent). At least 200 ng of DNase-treated total RNA with an RNA integrity number greater than 6 was used for rRNA depletion and generation of cDNA libraries via the ScriptSeq complete (bacterial) kit (Illumina/Epicentre) following the manufacturer’s protocol. Library quality was assessed using the 2100 Bioanalyzer (Agilent), and libraries were quantitated using KAPA library quantification kits (KAPA Biosystems). Pooled libraries were subjected to 75-bp paired-end sequencing according to the manufacturer’s protocol (Illumina HiSeq3000). Bcl2fastq2 conversion software (Illumina) was used to generate demultiplexed Fastq files.

### RNA sequencing analysis.

RNA sequencing data were trimmed to remove all bases below Q3 and had adapter sequence removed, using FaQCs (66). Trimmed sequences were aligned to the Staphylococcus aureus subsp. *aureus* strain Newman reference genome (accession number GCF_000010465.1) using BWA (67) and default alignment options. Read counts were generated using HT-Seq v0.6.1p1 (68) and default options. The EdgeR (69) package for R was used to analyze count files to identify differential gene expression (DGE). Read counts per gene were also analyzed and visualized using the Degust public server, Version 2.1, degust.erc.monash.edu. Raw read CSV files were uploaded to the server, analysis performed with the built-in Voom/Limma settings, the FDR cutoff modified to < 0.01, and the log fold change cutoff changed to 2. COG designations for individual genes were manually curated from microbesonline.org.

### Inductively coupled plasma mass spectrometry.

Four biological replicate overnight cultures of S. aureus Newman were diluted 1:100 into 5 ml TSB in 15 ml conical tubes and grown to mid-exponential phase for 3 h at 37°C with 180-rpm shaking. Cells were pelleted at 3,220 × *g* for 10 min at room temperature, supernatants were discarded, and cells were resuspended in 1 ml TSB containing 100 μM VU0026921, 1 mM MnCl_2_, 100 μM EDTA, 100 μM VU0026921 + 1 mM MnCl_2_, or 100 μM VU0026921 + 100 μM EDTA. Following incubation at 37°C with 180-rpm shaking for 30 min, cells were harvested by centrifugation at 3,220 × *g* for 10 min at 4°C and washed with 10 ml PBS (Corning, Corning, NY). Pellets were digested in 200 μl HNO_3_ (Optima grade metal-free; Fisher, Waltham, MA) and 50 μl hydrogen peroxide (ultratrace grade; Sigma) at 65°C overnight. Before analysis, 2 ml of UltraPure water was added to each sample. Elemental quantification of acid-digested samples was performed using an Agilent 7700 inductively coupled plasma mass spectrometer (Agilent, Santa Clara, CA) attached to a Teledyne CETAC Technologies ASX-560 autosampler (Teledyne CETAC Technologies, Omaha, NE). The following settings were fixed for the analysis: cell entrance = −40 V, cell exit = −60 V, plate bias = −60 V, OctP bias = −18 V, and collision cell helium flow = 4.5 ml/min. Optimal voltages for Extract 2, Omega Bias, Omega Lens, OctP RF, and Deflect were determined empirically before each sample set was analyzed. Element calibration curves were generated using Aristar ICP standard mix (VWR, Radnor, PA). Samples were introduced by peristaltic pump with 0.5-mm-internal-diameter tubing through a MicroMist borosilicate glass nebulizer (Agilent). Samples were initially up taken at 0.5 revolutions per second (rps) for 30 s followed by 30 s at 0.1 rps to stabilize the signal. Samples were analyzed in spectrum mode at 0.1 rps, collecting three points across each peak and performing three replicates of 100 sweeps for each element analyzed. Sampling probe and tubing were rinsed for 20 s at 0.5 rps with 2% nitric acid between every sample. Levels of ^34^S, ^59^Co, ^63^Cu, ^66^Zn, ^55^Mn, and ^56^Fe were measured, concentrations were determined utilizing a standard curve for each metal, and results were normalized by the concentration of ^34^S. Data were acquired and analyzed using the Agilent Mass Hunter workstation software version A.01.02.

### Reactive oxygen species quantification with dihydrorhodamine 123.

S. aureus Newman overnight cultures were diluted 10-fold into 150 μl TSB in black-walled 96-well plates containing (Fig. 3F) 50 μM DHR123 (Thermo Fisher, Waltham, MA) and vehicle, 50 μM ‘921, 1 mM MnCl_2_, or 50 μM ‘921 + 1 mM MnCl_2_ or (Fig. 4E and G) vehicle, 50 μM ‘921, 100 μM CoCl_2_ or CuSO_4_, or 50 μM ‘921 + 100 μM CoCl_2_ or CuSO_4_. Plates were incubated with shaking linearly at 37°C for 8 h on a BioTek Cytation 5 (BioTek, Winooski, VT). Every 30 min, the optical density was measured at 600 nm to determine bacterial growth and DHR123 fluorescence (excitation = 507 nm, emission = 529 nm) was measured to determine cellular ROS levels. ROS levels were normalized to OD_600_ to account for differences in S. aureus viability.

### Spectrophotometric determination of VU0026921 metal binding.

Zero or 100 μM ‘921 was combined with 0 or 500 μM CoCl_2_, CuSO_4_, MgCl_2_, CaCl_2_, MnCl_2_, FeSO_4_, or ZnCl_2_ in 200 μl water at 25°C. Absorbance spectra were taken from 280 nm to 600 nm in 10-nm increments on a BioTek Cytation 5 at 0, 5, 15, 30, 60, and 120 min following plating. Final spectra are metal spectra alone subtracted from ‘921 + metal spectra.

### Fatty acid biosynthesis inhibition experiments.

Approximately 10^5^ CFU of indicated bacteria were inoculated into 1 ml liquid medium (TSB) containing 2-fold dilutions of indicated antibiotics with or without oleic acid (18:1) supplemented at 40 μg/ml (S. aureus) or 10 μg/ml (E. faecalis) and incubated 20 h with shaking overnight in 13- by 100-mm glass test tubes at 37°C. The lowest dilution of antibiotics preventing visible growth of S. aureus was recorded as the MIC.

### Statistical analyses.

All raw numerical data were saved in Excel files and imported into GraphPad Prism for statistical analysis. All graphs are the result of a single representative experiment performed in biological triplicate, and all experiments were repeated to confirm results.

### Data availability.

The data received from RNA sequencing experiments have been deposited into the NCBI Gene Expression Omnibus (accession no. GSE128062). The portion of the data set which compares S. aureus treated with and without 1 mM MnCl_2_ has been previously published (34) and is also accessible under GEO accession number GSE124285.

10.1128/mBio.02555-20.10 TABLE S7 Primers used in this study. Download Table S7, XLSX file, 0.01 MB. Copyright © 2020 Juttukonda et al. 2020 Juttukonda et al. This content is distributed under the terms of the Creative Commons Attribution 4.0 International license.
