# Supplementary figures and images for: A Small-Molecule Modulator of Metal Homeostasis in Gram-Positive Pathogens
Source: mBio. 2020 Oct 27;11(5):e02555-20. doi: 10.1128/mBio.02555-20 (PMC7593973; doi:10.1128/mBio.02555-20)

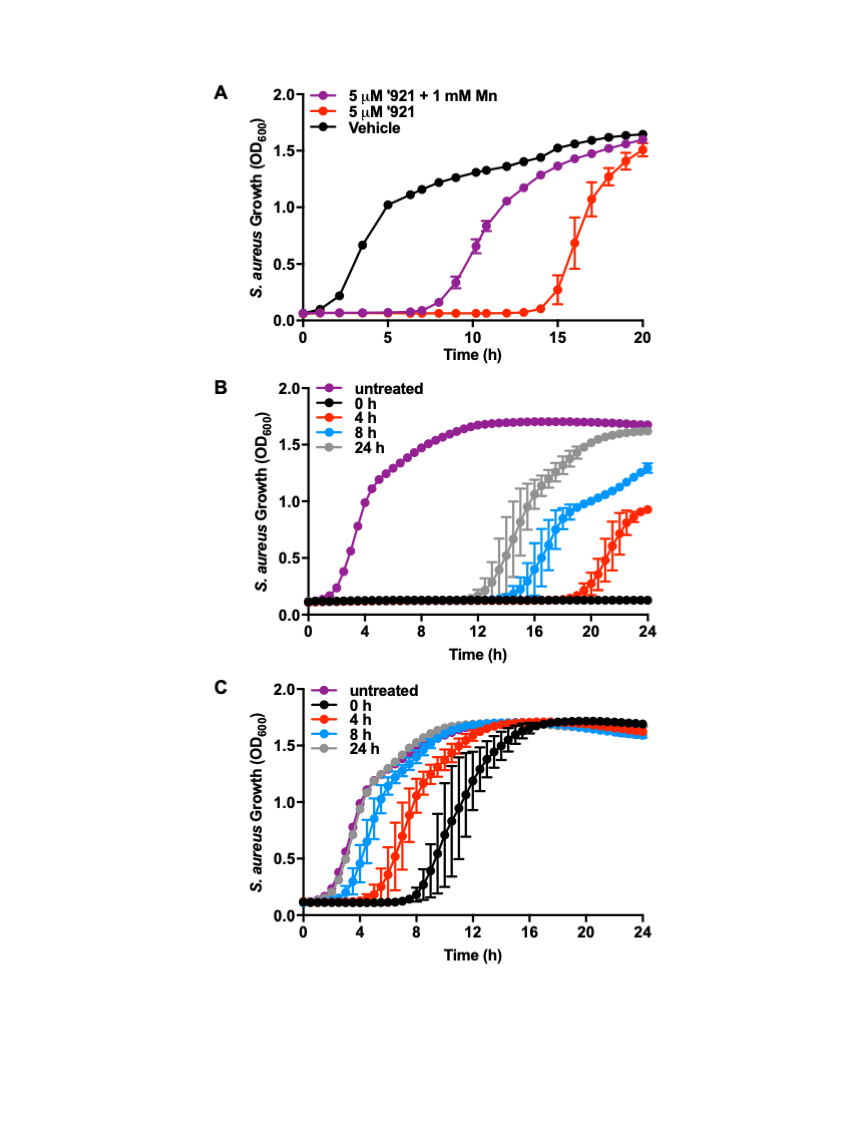

Supplement: FIG S1 [file mBio.02555-20-sf001.tif]

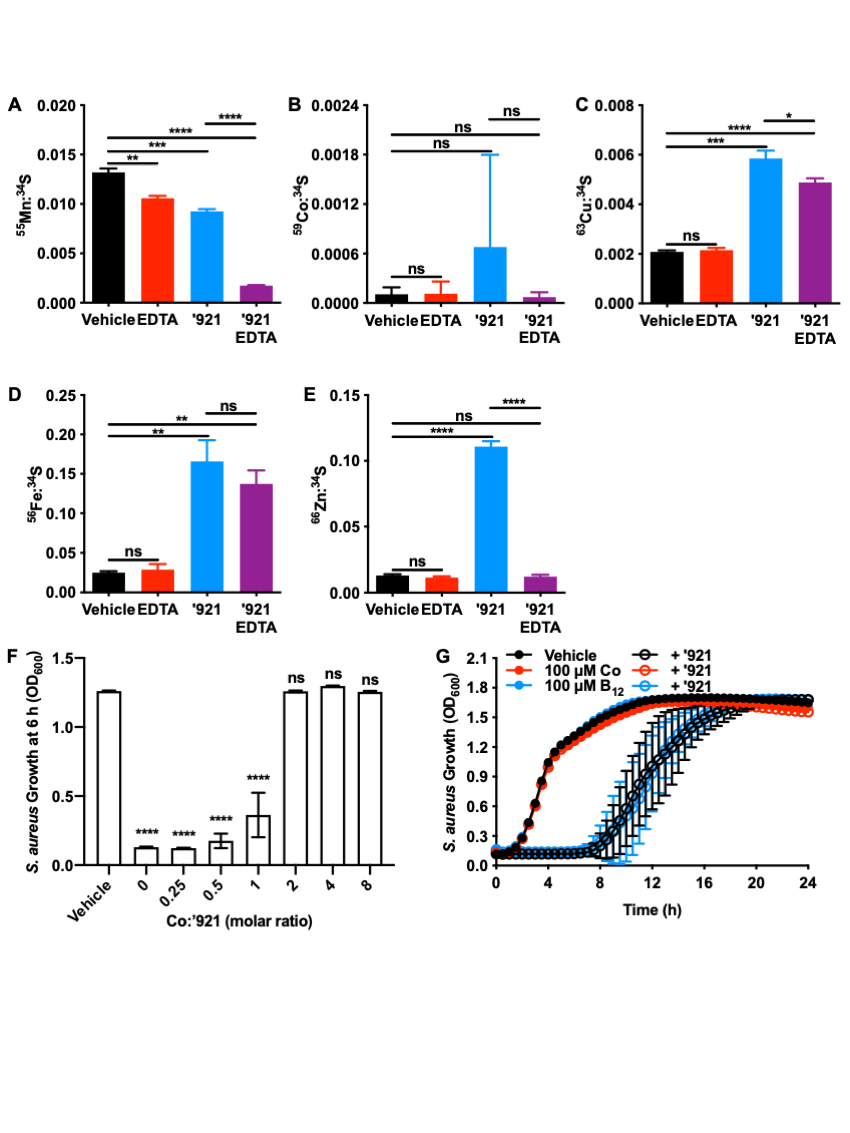

Supplement: FIG S2 [file mBio.02555-20-sf002.tif]

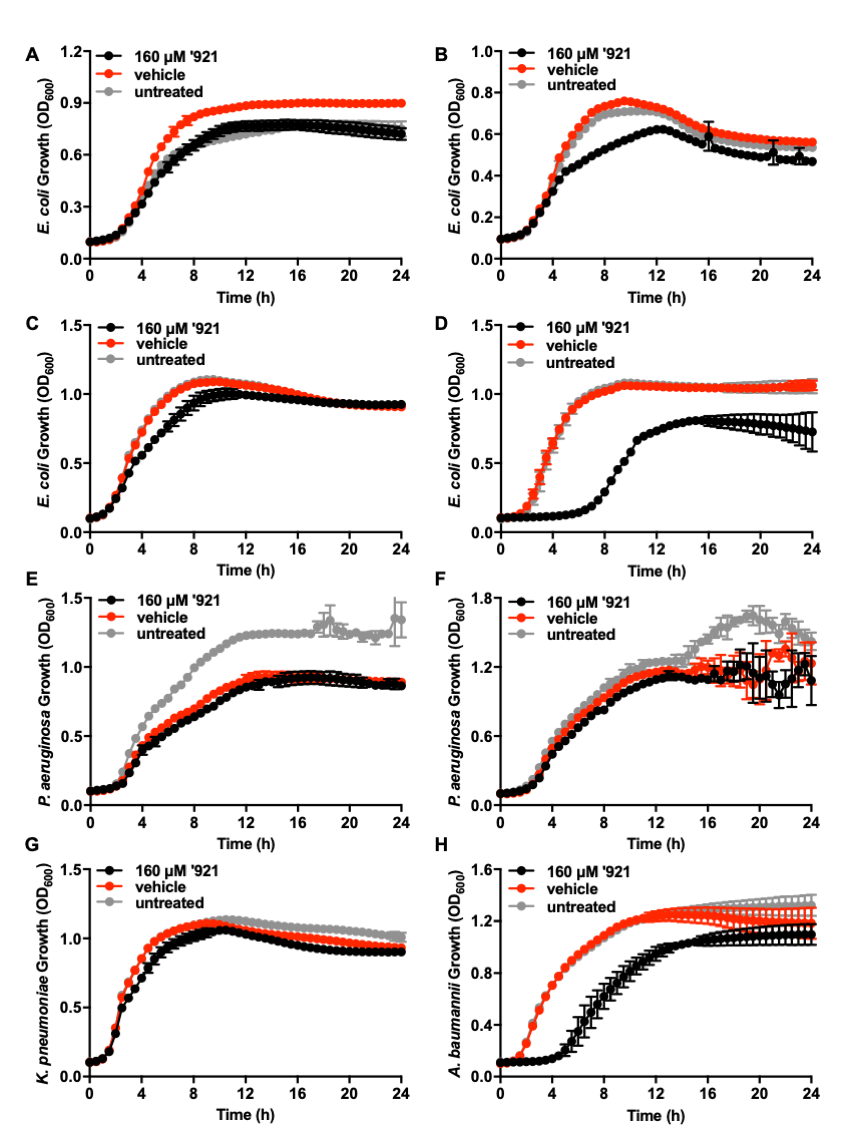

Supplement: FIG S3 [file mBio.02555-20-sf003.tif]
